# Screening analysis of ubiquitin ligases reveals G2E3 as a potential target for chemosensitizing cancer cells

**DOI:** 10.18632/oncotarget.2710

**Published:** 2014-12-15

**Authors:** Franziska Schmidt, Meike Kunze, Ann-Christine Loock, Matthias Dobbelstein

**Affiliations:** ^1^ Institute of Molecular Oncology, Göttingen Centre of Molecular Biosciences (GZMB), Faculty of Medicine, University of Göttingen, 37077 Göttingen, Germany

**Keywords:** DNA damage, cisplatin, gemcitabine, siRNA screen, ubiquitin ligase, G2E3, Chk1

## Abstract

Cisplatin is widely used against various tumors, but resistance is commonly encountered. By inducing DNA crosslinks, cisplatin triggers DNA damage response (DDR) and cell death. However, the molecular determinants of how cells respond to cisplatin are incompletely understood. Since ubiquitination plays a major role in DDR, we performed a high-content siRNA screen targeting 327 human ubiquitin ligases and 92 deubiquitinating enzymes in U2OS cells, interrogating the response to cisplatin. We quantified γH2AX by immunofluorescence and image analysis as a read-out for DNA damage. Among known mediators of DDR, the screen identified the ubiquitin ligase G2E3 as a new player in the response to cisplatin. G2E3 depletion led to decreased γH2AX levels and decreased phosphorylation of the checkpoint kinase 1 (Chk1) upon cisplatin. Moreover, loss of G2E3 triggered apoptosis and decreased proliferation of cancer cells. Treating cells with the nucleoside analogue gemcitabine led to increased accumulation of single-stranded DNA upon G2E3 depletion, pointing to a defect in replication. Furthermore, we show that endogenous G2E3 levels in cancer cells were down-regulated upon chemotherapeutic treatment. Taken together, our results suggest that G2E3 is a molecular determinant of the DDR and cell survival, and that its loss sensitizes tumor cells towards DNA-damaging treatment.

## INTRODUCTION

Cancer cells are sensitive to genotoxic stress, at least in part due to a loss of checkpoints that would otherwise prevent the unscheduled proliferation of cells that suffered DNA damage [reviewed in 1, 2]. Chemotherapy and radiotherapy take advantage of this sensitivity. Most DNA-damaging chemotherapeutics cause replication defects during S phase. When cells try to replicate damaged DNA, stalled and collapsed replication forks ultimately result in DNA double-strand breaks (DSBs), often associated with cell death.

Common DNA-damaging drugs include platinating agents which are used in the clinics since the 1970s to treat different types of cancer like testicular, ovarian, cervical, head and neck, lung and colorectal cancer [3, 4]. Cisplatin, along with oxaliplatin and carboplatin, is one of the most frequently prescribed chemotherapeutics for treating solid tumors. It induces intra- and interstrand crosslinks of the DNA, resulting in the accumulation of single-stranded DNA and DNA DSBs [3, 5]. Remarkably, although cisplatin has been used for many years, the cellular and molecular responses to it are incompletely understood. In particular, the mechanisms of tumor cell resistance are of high clinical relevance. We still lack detailed understanding of how DNA damage by cisplatin treatment is transduced to a signaling cascade and which components determine whether this response leads to cell cycle arrest, repair, or apoptosis. Therefore, our aim was to find new regulators of the DNA damage response (DDR) to cisplatin and potential targets whose inhibition could sensitize cancer cells to chemotherapeutic treatment.

Ubiquitin ligases and deubiquitinating enzymes (DUBs) play a major role in the DDR. Examples include the ubiquitin ligases RNF8 and RNF168 that mediate the response to DSBs [6–8]. BRCA1 is another important ubiquitin ligase with different roles in the DDR, in DNA repair and in cell cycle checkpoint control [reviewed in 9]. Considering the importance of the ubiquitin system in the DDR and open questions in the response to cisplatin treatment, we performed an siRNA screen and depleted cells of human ubiquitin ligases and DUBs. High-content siRNA screening had previously been used to identify components of the DDR and DNA repair [10, 11]. This was also done in the context of cisplatin treatment, e.g. screening the kinome to identify kinases in the response to cisplatin treatment of ovarian cancer cells [12]. Phosphorylation of the histone variant H2AX at Ser139 (then named γH2AX) was often detected as a marker of DDR in siRNA screens [e.g. 10, 13], but not in the context of cisplatin treatment and the ubiquitin system. Here, we quantified γH2AX to identify ubiquitin ligases and DUBs that modulate the DDR upon cisplatin treatment, and numerous known and hitherto unknown factors were found.

In particular, we identified the ubiquitin ligase G2E3 as a novel modulator of the response to DNA-damaging treatment. “G2E3” stands for “G2-specific E3 ligase” since it was originally reported as a putative ubiquitin ligase with maximum mRNA levels in the G2/M phase of the cell cycle [14]. The present work then aimed at understanding how G2E3 is involved in the cellular response to DNA damage. We observed that G2E3 depletion decreased the phosphorylation of H2AX as well as the checkpoint kinase 1 upon cisplatin treatment. Furthermore, loss of G2E3 augmented p53 accumulation and apoptosis. When treating cells with the nucleoside analogue gemcitabine, together with G2E3 depletion, the accumulation of single-stranded DNA was increased, suggesting that DNA replication was hindered. Finally, endogenous G2E3 levels in tumor cells were reduced upon chemotherapy. In summary, we introduce G2E3 as a novel modulator of the DDR whose loss sensitizes cancer cells towards DNA damage.

## RESULTS

### A high-content siRNA screen identifies modulators of the DDR to cisplatin, including G2E3

**Figure 1:**
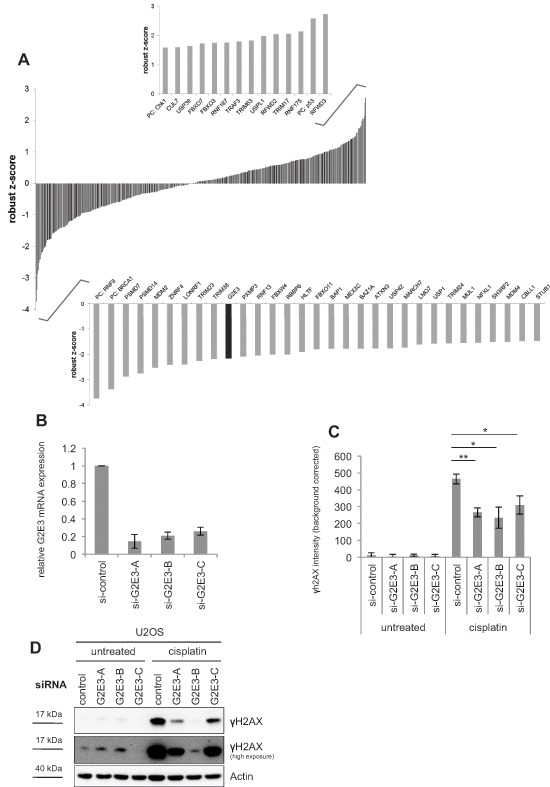
A high-content siRNA screen identifies modulators of the DDR to cisplatin, including G2E3 **(A) A high-content siRNA screen to determine the impact of human ubiquitin ligases and deubiquitinating enzymes (DUBs) on cisplatin-induced H2AX phosphorylation**. U2OS cells were transfected with siRNAs targeting 327 human ubiquitin ligases and 92 DUBs, using three different siRNAs per gene. The cells were then treated with 30 μM cisplatin for 16 h, fixed and stained for γH2AX. Automated microscopy and image analysis was performed using the BD Pathway System. A robust z-score was assigned to each siRNA as a measure of H2AX phosphorylation. Candidates with significant increase (positive robust z-score) and decrease (negative robust z-score) in γH2AX fluorescence intensity are depicted as average robust z-score of three siRNAs per gene. G2E3 is marked in black. PC = positive control. **(B) Knockdown efficiency of G2E3 siRNAs.** U2OS cells were transfected with three different siRNAs against G2E3 (same siRNAs as in the screen) and harvested 64 h after siRNA transfection. G2E3 mRNA levels were analyzed by quantitative RT-PCR and normalized to the reference gene GAPDH. Data are represented as mean. Error bars represent the standard deviation (SD, *n* = 3). **(C) Knockdown of G2E3 decreases the phosphorylation of H2AX in U2OS cells after cisplatin treatment.** U2OS cells were transfected with three different siRNAs against G2E3. The cells were either left untreated or treated with 30 μM cisplatin for 16 h, fixed and stained for γH2AX, followed by automated microscopy and image analysis. Results were corrected for background fluorescence. Data are represented as mean. Error bars represent the standard deviation (SD, *n* = 3). *p < 0.05, **p < 0.01 (Student's t-test). **(D) Knockdown of G2E3 decreases γH2AX accumulation, as determined by immunoblot analysis.** U2OS cells were depleted of G2E3 by siRNA-mediated knockdown. Where indicated, the cells were treated with 30 μM cisplatin for 16 h. Cell lysates were analyzed by immunoblotting and detection of γH2AX.

To identify new mediators of the cellular response to cisplatin, U2OS cells were transfected with a collection of siRNAs targeting 327 human ubiquitin ligases and 92 deubiquitinating enzymes (DUBs). The osteosarcoma cell line U2OS was chosen for better comparison with previous studies on the DDR that used the same cell line in immunofluorescence microscopy [8, 11, 15]. Furthermore, chemotherapy for osteosarcoma patients includes cisplatin treatment [16]. After transfection, U2OS cells were treated with cisplatin, fixed and subsequently stained for γH2AX as a marker of the DDR. The extent of γH2AX accumulation was quantified by automated microscopy and image analysis. As a statistical measure of H2AX phosphorylation, a robust z-score was assigned to each siRNA. Fig. 1A shows candidates with substantial increase (positive robust z-score) or decrease (negative robust z-score) in γH2AX fluorescence intensity. For primary results of the high-content siRNA screen, please see Tab. S1. As expected, our screen identified gene products known to be involved in the DDR and DNA repair, but also in the p53-pathway. Knockdown of **Mdm2** and knockdown of three regulators of the p53-Mdm2-pathway, namely **Mdm4** (Mdmx), **RBBP6** (Retinoblastoma binding protein 6) and **STUB1** (CHIP) resulted in alleviated H2AX phosphorylation. Mdmx heterodimerizes with Mdm2 to repress p53 transactivation activity [17]. RBBP6 interacts with and activates Mdm2 [18]. The ubiquitin ligase STUB1 (CHIP) targets p53 for degradation [19]. Knockdown of these p53 regulators would lead to accumulation of p53 and thus induce cell cycle arrest via CDKN1A/p21 [20]. Such an arrest in the G1 phase of the cell cycle represents at least one plausible mechanism to avoid replicative stress and thus DNA damage.

The screen also identified the deubiquitinating enzyme **USP1** (ubiquitin-specific protease 1) which deubiquitinates FANCD2, a protein involved in the Fanconi anemia DNA repair pathway [21]. USP1 is also involved in translesion synthesis (TLS) of DNA by deubiquitinating PCNA [22]. Furthermore, we found two proteasomal subunits, **PSMD7** and **PSMD14** (26S proteasome non-ATPase regulatory subunit 7 and 14) to be required for full response to cisplatin treatment. In agreement, the proteasomal deubiquitinating enzyme PSMD14 (also called POH1) has been shown to negatively regulate the RNF8-dependent response to DNA DSBs [23]. The identification of known transmitters of the DDR and p53-pathway validates the screen. Notably, the procedure also identified a putative ubiquitin ligase, G2E3, as a transmitter of the DDR in this context. G2E3 was previously characterized as an essential gene product for murine development, and as a determinant of cell fate [24], but not DNA damage signaling. These features made G2E3 an interesting candidate for further investigation. G2E3 knockdown led to decrease in γH2AX levels after cisplatin treatment as detected by immunofluorescence (Fig. 1A). The knockdown of G2E3 with three different siRNAs was confirmed by quantitative RT-PCR (Fig. 1B), and decreased H2AX phosphorylation in cisplatin-treated U2OS cells was confirmed by immunofluorescence staining (Fig. 1C) and immunoblot analysis (Fig. 1D). Thus, G2E3 is required for transmitting the DDR signal on H2AX in cisplatin-treated cells.

### G2E3 depletion induces p53-dependent accumulation of p21

**Figure 2:**
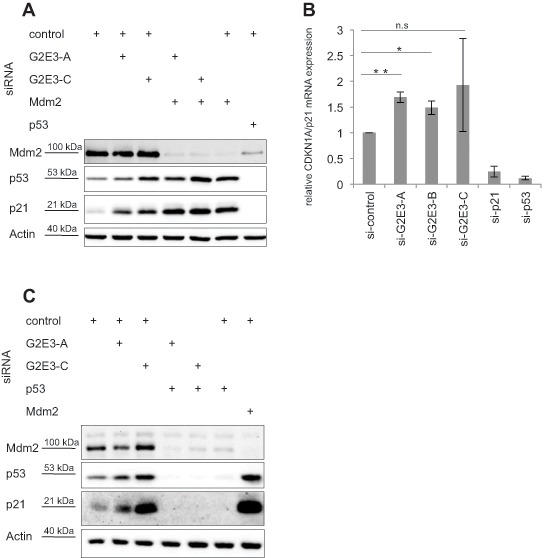
G2E3 depletion induces p53-dependent accumulation of p21 **(A) G2E3 depletion enhances the levels of p21.** U2OS cells were transfected with combinations of siRNAs targeting G2E3 and Mdm2 as indicated. Knockdown of p53 served as a control. After 48 h, the cells were harvested and analyzed by immunoblotting and detection using antibodies to the indicated proteins. **(B) G2E3 knockdown induces CDKN1A/p21 mRNA accumulation.** U2OS cells were depleted of G2E3, p21 and p53 by siRNA-mediated knockdown. After 64 h, the cells were harvested and CDKN1A/p21 mRNA levels were analyzed by quantitative RT-PCR, in relation to the reference gene GAPDH. Data are represented as mean. Error bars represent the standard deviation (SD, *n* = 3). *p < 0.05, **p < 0.01, n.s. = not significant (Student's t-test). **(C) p21 induction by G2E3 depletion depends on p53.** U2OS cells were transfected with combinations of siRNAs targeting G2E3 and p53 as indicated. Knockdown of Mdm2 served as control. After 64 h, cells were harvested and analyzed as in A.

Since the knockdown of the aforementioned p53 regulators led to decreased H2AX phosphorylation, we investigated whether G2E3 depletion affects p53 and p21 levels as well. Indeed, analysis by immunoblotting revealed that p53 and p21 levels were augmented upon G2E3 knockdown in untreated U2OS cells (Fig. 2A). Knockdown of Mdm2 served as positive control, causing p53 induction and p21 expression. Similarly, p21 mRNA levels were induced upon G2E3 knockdown (Fig. 2B). We also performed a double-knockdown of Mdm2 and G2E3, but did not observe additive p21 accumulation (Fig. 2A), arguing that G2E3 and Mdm2 act on p53 activity in an epistatic fashion. In contrast, a double-knockdown of G2E3 and p53 abolished p21 induction (Fig. 2C), strongly suggesting that G2E3 knockdown induces p21 through p53. Taken together, these results identify G2E3 as a negative regulator of p53 activity.

### Depletion of G2E3 decreases the proliferation rate of cancer cells

**Figure 3:**
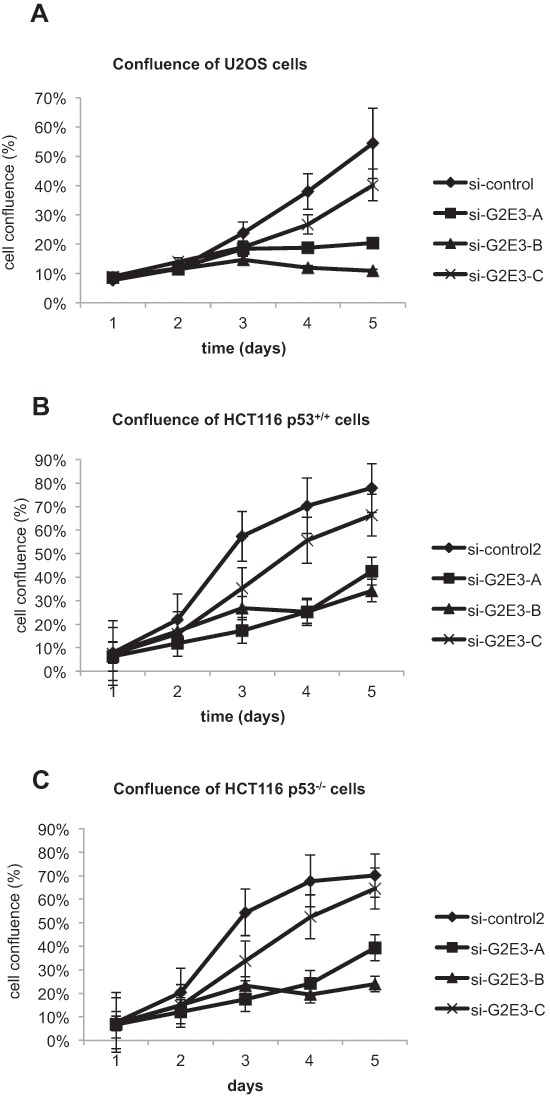
Depletion of G2E3 decreases the proliferation rate of cancer cells U2OS **(A)**, HCT116 p53^+/+^ **(B)** and HCT116 p53^−/−^ **(C)** cells were depleted of G2E3 by siRNA-mediated knockdown with three different siRNAs. Cell proliferation was determined by measuring cell confluence on five consecutive days by bright-field microscopy, using a Celigo cell cytometer (Cyntellect/Brooks Automation). Data are represented as mean. Error bars represent the standard error of the mean (SEM, *n* = 3 for U2OS and *n* = 4 for HCT116).

Next, we evaluated the role of G2E3 in cell survival by continuously monitoring cell confluence after siRNA transfection. After G2E3 knockdown in U2OS (osteosarcoma) and HCT116 p53^+/+^ (colon carcinoma) cells, we found that the amount of surviving and proliferating cells was decreased (Fig. 3A, 3B). Surprisingly, this was also found in HCT116 p53^−/−^ cells (Fig. 3C). Hence, while G2E3 is required to suppress p53 (Fig. 2), it also sustains clonogenic survival by p53-independent mechanisms.

### Removing G2E3 results in p53-independent apoptosis

**Figure 4:**
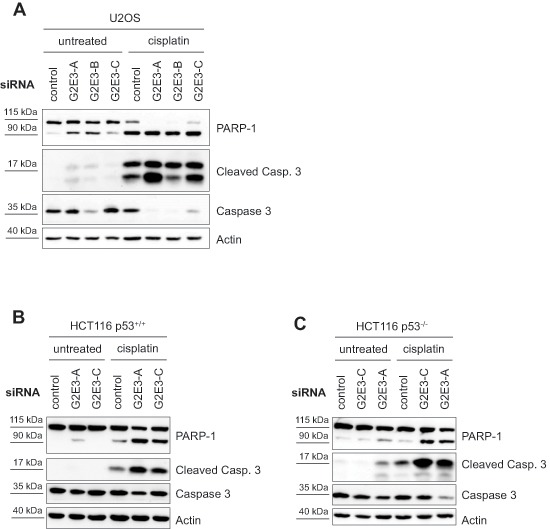
Knockdown of G2E3 results in p53-independent apoptosis and chemosensitization of osteosarcoma and colon carcinoma cells U2OS **(A)**, HCT116 p53^+/+^ **(B)** and HCT116 p53^−/−^ **(C)** cells were depleted of G2E3 by siRNA-mediated knockdown and either left untreated or treated with 30 μM cisplatin for 16 h. Cell lysates were analyzed by immunoblotting. Markers for apoptosis are cleaved caspase 3 (Cleaved Casp. 3), in comparison with total caspase 3, and cleaved PARP-1 (full-length protein at 115 kDa and cleaved PARP-1 at 90 kDa).

Since the amount of proliferating cells was decreased upon G2E3 knockdown, we tested whether this correlates with increased susceptibility to apoptosis. To this end, we depleted U2OS cells of G2E3, followed by cisplatin treatment and immunoblot analysis of caspase substrates. Indeed, apoptosis was accelerated upon G2E3 knockdown, as revealed by the detection of cleaved caspase 3 and cleaved PARP-1 (Fig. 4A). Even in otherwise untreated cells, induction of apoptosis upon G2E3 knockdown was observed, and this was further enhanced by cisplatin. Knockdown of G2E3 also led to increased apoptosis in the colon carcinoma cell line HCT116 (Fig. 4B), and strikingly, also in p53-deficient HCT116 cells (Fig. 4C). This observation suggests that G2E3 plays a p53-independent role in avoiding apoptosis. These results are in line with the embryonic lethality of G2E3 knock-out mice as well as G2E3/p53 double knock-out mice, which die due to apoptosis and involution of the blastocyst [24]. In summary, the removal of G2E3 leads to increased apoptosis in various cell lines in a p53-independent manner, pointing to a pro-survival role of this gene product.

### G2E3 knockdown attenuates ATR-Chk1 signaling in cisplatin-treated cells

To investigate how G2E3 affects γH2AX accumulation and cell survival, we explored its impact on the DDR itself. During the DDR, damaged DNA is recognized by kinases of the PIKK (phosphatidylinositol 3-kinase-related kinase) family, including ATM (ataxia telangiectasia mutated) and ATR (ATM and Rad3-related). Double-stranded DNA activates ATM and the checkpoint kinase Chk2, while replicative stress and single-stranded DNA (ssDNA) trigger the ATR-Chk1 pathway. ATR is found in a complex with ATRIP (ATR-interacting protein) [25] and thereby capable of phosphorylating substrates like the checkpoint kinase Chk1 [26, 27]. Cisplatin induces crosslinks between DNA strands, forming obstacles to transcription and replication, and giving rise to replicative stress [3]. Stalled or collapsed replication forks activate the ATR-Chk1 pathway. Secondary DNA DSBs presumably activate the ATM-Chk2 pathway.

**Figure 5:**
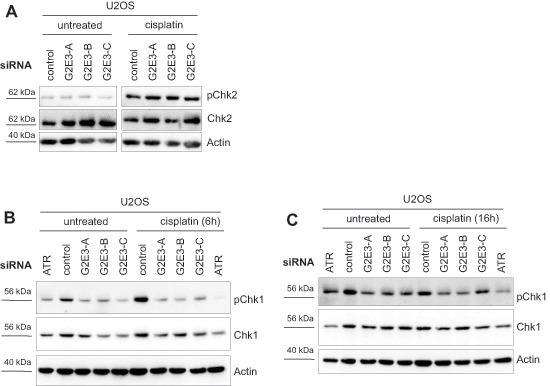
Decrease in phospho-Chk1 levels after G2E3 knockdown and cisplatin treatment **(A) G2E3 knockdown does not affect phospho-Chk2 levels upon cisplatin treatment.** U2OS cells were depleted of G2E3 by siRNA-mediated knockdown and either left untreated or treated with 30 μM cisplatin for 8 h. Cell lysates were analyzed by immunoblotting and detection of phospho-Chk2 (Thr68) and total Chk2 levels. Actin staining served as loading control. **(B) Knockdown of G2E3 decreases the phosphorylation of Chk1 in U2OS cells after cisplatin treatment.** U2OS cells were depleted of G2E3 by siRNA-mediated knockdown. Cells were either left untreated or treated with 30 μM cisplatin for 6 h. Cell lysates were analyzed by immunoblotting and detection of phospho-Chk1 (Ser317) and Chk1. Actin staining served as loading control. **(C) Decrease in Chk1 phosphorylation after G2E3 knockdown depends on duration of cisplatin treatment.** Cells were treated as in B, but with 30 μM cisplatin for 16 h.

We investigated whether G2E3 knockdown affects ATM and/or ATR signaling and observed that phospho-Chk2 (Thr68) levels were not changed upon G2E3 knockdown and cisplatin treatment (Fig. 5A). Instead, we found that removing G2E3 decreased the phosphorylation of Chk1 (Ser317) in U2OS cells after cisplatin treatment (Fig. 5B, 5C). This effect was stronger after a short exposure to cisplatin (for 6 h), but still detectable after a longer exposure (for 16 h). Thus, G2E3 is required to enable the activation of Chk1, but not Chk2, in response to cisplatin treatment.

### G2E3 knockdown inhibits Chk1 activation and increases the accumulation of single-stranded DNA after gemcitabine treatment

**Figure 6:**
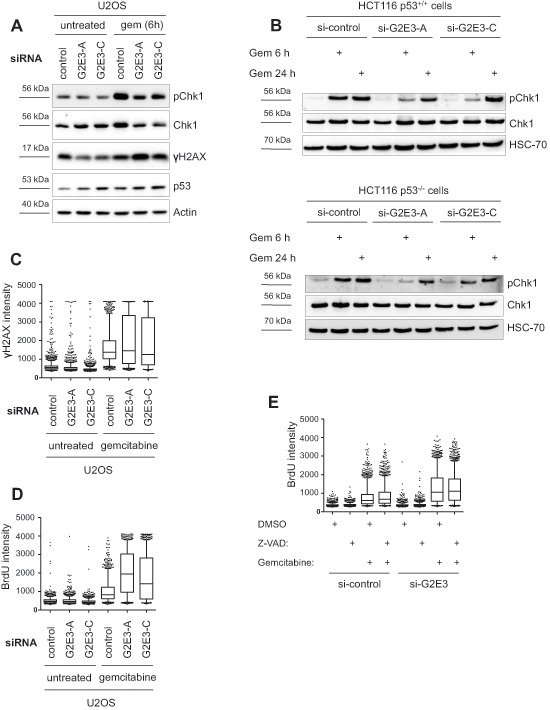
Decrease in phospho-Chk1 levels and increased accumulation of single-stranded DNA after G2E3 knockdown and gemcitabine treatment **(A) Knockdown of G2E3 decreases phosphorylation of Chk1 in U2OS cells after gemcitabine treatment.** U2OS cells were depleted of G2E3 by siRNA-mediated knockdown. Cells were either left untreated or treated with 300 nM gemcitabine for 6 h. Cell lysates were analyzed by immunoblotting and detection of phospho-Chk1 (Ser317), Chk1, p53 and γH2AX. **(B) Knockdown of G2E3 decreases phosphorylation of Chk1 in HCT116 p53^+/+^ and HCT116 p53^−/−^ cells shortly after gemcitabine treatment**. HCT116 p53^+/+^ and HCT116 p53^−/−^ cells were depleted of G2E3 by siRNA-mediated knockdown. Cells were either left untreated or treated with 300 nM gemcitabine for 6 and 24 h. Cell lysates were analyzed by immunoblotting and detection of phospho-Chk1 (Ser317) and Chk1. **(C) Knockdown of G2E3 increases phosphorylation of H2AX in U2OS cells after gemcitabine treatment.** U2OS cells were transfected with two different siRNAs against G2E3. Cells were either left untreated or treated with 300 nM gemcitabine for 24 h, fixed and stained for γH2AX. Automated microscopy and image analysis was performed using the BD Pathway System. The results shown are representative of three independent replicates. Box plots represent median (black line), first and third quartiles (box) and 5 and 95% percentiles (whiskers). **(D) G2E3 inhibition increases the accumulation of ssDNA after gemcitabine treatment.** U2OS cells were transfected with two different siRNAs against G2E3 and labeled with BrdU for 24 h. Cells were either left untreated or treated with 300 nM gemcitabine for 24 h, under continued presence of BrdU, fixed and processed for ssDNA quantification by immunofluorescent detection of accessible BrdU, without denaturing of the DNA. The results shown are representative of three independent replicates. Box plots represent median (black line), first and third quartiles (box) and 5 and 95% percentiles (whiskers). **(E) Increased accumulation of ssDNA after G2E3 knockdown and gemcitabine treatment does not depend on caspase activity**. U2OS cells were transfected with siRNA against G2E3 and labeled with BrdU for 24 h. Subsequently, cells were either left untreated or treated with 300 nM gemcitabine and/or 50 μM caspase inhibitor Z-VAD (DMSO as control) for 24 h, under continued presence of BrdU. Cells were fixed and processed for ssDNA quantification as in D.

Chk1 activation is most relevant to replicative stress during S phase. We therefore studied the impact of G2E3 knockdown on the cellular response to an inducer of replicative stress, i.e. gemcitabine. Gemcitabine is a nucleoside analogue which perturbs replication by getting incorporated into DNA instead of dCTP, resulting in stalled replication forks. Furthermore, gemcitabine inhibits ribonucleotide reductase, leading to imbalance of the dNTP pool. We found that phospho-Chk1 (Ser317) levels were decreased upon G2E3 knockdown in U2OS cells treated with gemcitabine (Fig. 6A). Decreased phospho-Chk1 levels upon G2E3 knockdown were also observed in HCT116 p53^+/+^ and HCT116 p53^−/−^ cells after gemcitabine treatment (Fig. 6B). When we further investigated the impact of G2E3 on the DDR to gemcitabine treatment, we found that G2E3 knockdown increased the fraction of cells that contain high γH2AX levels upon gemcitabine treatment (Fig. 6C). We hypothesized that this is due to increased replicative stress and therefore investigated gemcitabine-induced accumulation of single-stranded DNA (ssDNA) upon G2E3 knockdown. Accumulation of ssDNA is due to perturbed replication but ongoing helicase activity at replication forks. This can be detected by immunofluorescent staining of exposed BrdU-labelled DNA strands [28]. We found that cells in which G2E3 was depleted accumulated far more ssDNA in response to gemcitabine treatment than control cells (Fig. 6D). This accumulation was still observed in the presence of a caspase inhibitor, Z-VAD (Fig. 6E), arguing that it is not a secondary effect of apoptosis. Taken together, these results suggest that G2E3 depletion decreases ATR-Chk1 signaling and increases replicative stress in gemcitabine-treated cancer cells.

### Endogenous G2E3 mRNA and protein levels are decreased after DNA damage, independent of p53

**Figure 7:**
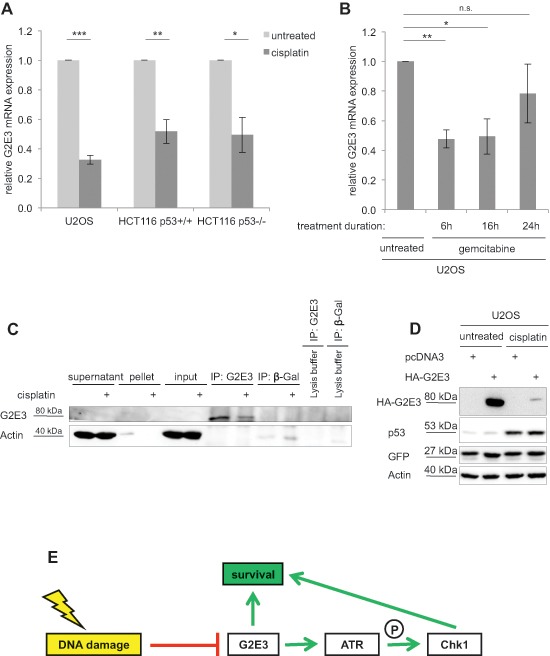
Decrease of endogenous G2E3 levels after DNA damage **(A) G2E3 mRNA levels are decreased upon cisplatin treatment.** U2OS, HCT116 p53^+/+^ and HCT116 p53^−/−^ cells were transfected with control-siRNA and either left untreated or treated with 30 μM cisplatin for 16 h. Cells were harvested and G2E3 mRNA levels were analyzed by quantitative RT-PCR and normalized to the reference gene GAPDH. Data are represented as mean. Error bars represent the standard deviation (SD, *n* = 3). *p < 0.05, **p < 0.01, ***p < 0.001 (Student's t-test). **(B) G2E3 mRNA levels are reduced by gemcitabine treatment.** U2OS cells were treated with 300 nM gemcitabine for 6, 16 or 24 h. The cells were harvested after a total incubation time of 48 h. G2E3 mRNA levels were analyzed as in A. **(C) Endogenous G2E3 protein levels are decreased after DNA damage.** U2OS cells were either left untreated or treated with 30 μM cisplatin for 16 h, and during the last 4 h with 20 μM of the proteasome inhibitor MG132. G2E3 was immunoprecipitated, followed by immunoblot detection of G2E3. β-Galactosidase (β-Gal) was used as control antibody. As a further control, lysis buffer without cell lysate was incubated with the indicated antibodies. **(D) HA-G2E3 protein levels are reduced by cisplatin treatment.** U2OS cells were transiently transfected with a plasmid encoding HA-G2E3 or with an empty plasmid (pcDNA3) as control, followed by treatment with 30 μM cisplatin for 16 h as indicated. Cell lysates were analyzed by immunoblotting and detection using antibodies to the indicated proteins. Cotransfection of a GFP expression plasmid served as control for transfection efficiency. **(E) Model of G2E3 affecting cell survival.** G2E3 is down-regulated in a DNA damage-responsive manner. On the other hand, G2E3 supports cell survival. In part, this pro-survival function is carried out by sustaining the ATR-Chk1 signaling pathway, thereby avoiding replicative stress.

G2E3 was initially identified by global gene expression profiling in HeLa cells based on its maximum mRNA levels in G2 phase [14]. We found this in synchronized U2OS cells as well (Fig. S7A). Furthermore, since G2E3 mRNA synthesis was reported to be decreased upon γ-irradiation in HeLa cells [14], we tested whether the same is true in response to chemotherapy. To this end, we treated U2OS, HCT116 p53^+/+^ and HCT116 p53^−/−^ cells with cisplatin and determined the G2E3 mRNA levels by quantitative RT-PCR. Indeed, we observed decreased G2E3 expression after cisplatin treatment (Fig. 7A). Likewise, treatment of U2OS cells with gemcitabine reduced G2E3 mRNA levels (Fig. 7B), as well as treatment with neocarzinostatin (Fig. S7B), suggesting general DNA damage-responsive down-regulation of G2E3. Since none of the available antibodies to G2E3 detected the protein in blots of cell lysates, we followed an immunoprecipitation-immunoblot protocol to quantify the levels of G2E3. This revealed that cisplatin decreased the levels of G2E3 protein as well (Fig. 7C). Furthermore, the amount of overexpressed, HA-tagged G2E3 (but not co-expressed GFP) was strongly decreased upon cisplatin treatment (Fig. 7D). These results suggest that G2E3 does not only regulate parameters within the DDR, but that it is itself regulated by DNA damage, apparently at transcriptional as well as posttranscriptional levels. It was reported that p53 can repress G2E3 transcription via the *large intergenic noncoding RNA* lincRNA-p21, a p53-target in response to DNA damage [29]. However, the reduction in G2E3 expression by cisplatin remained unchanged when depleting p53 (Fig. S7C). Accordingly, treatment of HCT116 p53^+/+^ and HCT116 p53^−/−^ cells with cisplatin reduced G2E3 to a similar extent (Fig. 7A). Taken together, DNA damage suppresses G2E3 levels in a manner that does not depend on p53.

## DISCUSSION

In this study, we identified G2E3, a putative ubiquitin ligase, as a new modulator of the DNA damage response and cell survival. We show that G2E3 is a negative regulator of p53 activity. The removal of G2E3 leads to increased apoptosis by p53-independent mechanisms, arguing for a pro-survival role of this protein. Furthermore, DNA damage reduces G2E3 levels in a p53-independent manner. Our results strongly suggest that G2E3 depletion alleviates ATR-Chk1 signaling and enhances replicative stress in cancer cells. Thus, we propose a model (Fig. 7E) according to that G2E3 acts as a pro-survival factor. In part, this function is carried out by sustaining the ATR-Chk1 signaling pathway, thereby avoiding replicative stress.

It has been shown previously that Chk1 depletion augments cell death upon treatment with replication inhibitors [30]. Thus, induction of apoptosis upon depletion of G2E3 and DNA-damaging treatment could be due to a decrease in phospho-Chk1 levels. This hypothesis is supported by the fact that besides G2E3 knock-out [24], apoptosis-dependent embryonic lethality has also been shown in Chk1 knock-out mice [26]. Apoptosis in Chk1^−/−^ blastocysts is p53-independent, just as in G2E3 knock-out cells, since double-knockout of Chk1 and p53 cannot rescue or delay early lethality in Chk1^−/−^ embryos [26]. Chk1 was first reported to be involved in signaling of single-stranded DNA, but furthermore has been shown to suppress apoptosis in response to replicative stress in both p53-proficient and p53-deficient cells [30]. So far, we could not identify the mechanism leading to decreased Chk1 phosphorylation upon G2E3 depletion. It is possible that proteins involved in the ATR-Chk1 pathway upstream of Chk1 are regulated by G2E3, but so far, the physiological target proteins of G2E3 have not been identified. The role of G2E3 could potentially be to ubiquitinate its substrate or to be involved in its regulation by protein-protein interaction. It was reported that overexpressed GFP-G2E3 localizes to the nucleus of several cell types, including Cos-7, SiHa and BSC-40 cells, and that G2E3 can undergo nucleocytoplasmic shuttling [31]. This is compatible with a role of G2E3 as a regulator of nuclear factors, e.g. p53 and Chk1. Different regulators affect the stability and activity of p53 by means of post-translational modifications (e.g. phosphorylation, acetylation, ubiquitination, sumoylation), protein-protein interactions and subcellular localization [reviewed in 32, 33, 34]. The main negative regulator of p53 function and stability is the RING E3 ligase Mdm2. Mdm2 and p53 interact at the N-terminal domain of p53, and this interaction inhibits the transactivation of genes by p53 [35, 36]. In addition, Mdm2 mediates the proteasomal degradation of p53 [37, 38]. So far, we do not have evidence for a direct regulation of p53 activity through G2E3 (e.g. by ubiquitination). Possibly, G2E3 can indirectly disrupt the interaction of p53 and Mdm2 in untreated cells, leading to the observed accumulation of p53 and p21.

We also found that the cellular response upon G2E3 depletion depends on the specific chemotherapeutic drug under study. G2E3 knockdown augments the DDR of cells to gemcitabine treatment (increase in γH2AX levels, Fig. 6A, 6C), whereas the DDR to cisplatin treatment is reduced (decrease in γH2AX levels, Fig. 1C, 1D). The reasons could be the different mechanisms of action of both reagents. Cisplatin induces intra- and interstrand crosslinks of the DNA, resulting in stalled replication forks and accumulation of single-stranded DNA [3, 5]. On the other hand, gemcitabine is a nucleoside analogue which disrupts replication by getting incorporated into DNA, resulting in stalled replication forks, too. But moreover, gemcitabine inhibits ribonucleotide reductase which leads to imbalance of the dNTP pool. Hence, the function of G2E3 seems to be dependent on the biochemical pathways involved. Of note, however, removing G2E3 decreased cell survival regardless of which drug was applied.

Since depletion of G2E3 decreases Chk1 activity, G2E3 inhibition may sensitize cancer cells in a similar way as Chk1 inhibition, a strategy which has been used in experimental cancer therapy as well as in early clinical studies [reviewed in 39, 40, 41]. Other strategies to interfere with Chk1 activation include the targeting of its natural regulators, especially ATR [26, 27]. Upon replicative stress, Chk1 stability is regulated by ubiquitination and degradation through E3 ligase complexes containing Cul1 or Cul4A [42] and by the Fbx6 (F box protein 6)-containing SCF (Skp1-Cul1-F box) E3 ligase [43]. G2E3 appears to represent an E3 ligase that specifically affects the levels of activated Chk1.

When exploring G2E3 expression in cancerous tissue using the GeneSapiens gene expression database [44], we found G2E3 expression to be substantially increased in testicular cancer, especially in seminoma. High G2E3 expression was also found in healthy human testis tissue and in testis of adult heterozygous G2E3 mice carrying a reporter driven by the endogenous G2E3 promoter [24]. Most testicular cancers respond well to cisplatin treatment, but therapeutic failures do occur. Our results suggest that initially high G2E3 levels and/or variations in the ability to degrade G2E3 in response to DDR may contribute to such failures. The fact that G2E3 is suppressed in its levels by cisplatin treatment raises the perspective of evaluating G2E3 as a predictive biomarker.

Furthermore, our findings bring up the question whether G2E3 represents a suitable anticancer drug target. In the past, two main strategies have been applied to design modulators of the ubiquitin system [45]. Firstly, small-molecule inhibitors have been developed in order to block the catalytic activity of enzymes (e.g. E1-activating enzyme inhibitors, E3 ubiquitin ligase inhibitors, proteasome inhibitors). Secondly, the interaction between ubiquitin ligases and substrates has been prevented. The advantage of inhibitors of protein-protein interactions is their increased specificity, but for applying it to G2E3, its substrates would first need to be identified. G2E3 consists of four domains that can act as E3 ligases (three domains with similarity to both RING and PHD domains and a fourth HECT domain) [31]. *In vitro* ubiquitin ligase activity was shown for two of the PHD/RING domains [24]. Thus, the PHD/RING domains in G2E3 may represent a suitable cancer drug target. Small molecules inhibiting a RING domain have been designed in the past. For instance, the compound HL198 targets the active site of the RING type E3 ligase Mdm2 [46] and this blocks p53 ubiquitination.

Platinating agents like cisplatin as well as other chemotherapeutics are limited from reaching their full potential due to resistance mechanisms and toxicities. It was reported for several cancer types that patients respond initially well to cisplatin therapy, but that the relapse rate is high. Examples include treatment of small cell lung carcinomas with a relapse rate of 95% [3] and treatment of head and neck cancers for which cisplatin is the first-line therapy. Here, the response rate is only 20–30% [47]. Reasons for resistance towards cisplatin include increased efflux or decreased influx, detoxification mechanisms (e.g. by glutathione), increased DNA repair (e.g. through activation of NER, MMR, and/or HR pathways) and bypassing lesions during replication – or a combination of these processes [3]. Factors involved in these pathways – such as G2E3 – represent potential targets for overcoming such resistance.

## METHODS

### Cell culture, transfection and treatments

U2OS cells were cultured in DMEM (Gibco/Life Technologies) supplemented with 10% FCS and antibiotics. siRNA-mediated knockdown was performed by reverse transfection of U2OS and HCT116 cells with 10 nM Silencer Select siRNAs (all Ambion/Life Technologies) using Lipofectamine 2000 (Invitrogen/Life Technologies). Plasmid transfection of U2OS cells was performed using Lipofectamine 2000 (Invitrogen/Life Technologies). For chemotherapeutic treatment, we used cisplatin (Teva), gemcitabine (Actavis) and neocarzinostatin (Sigma). For caspase inhibition, we used Z-VAD (Sigma) and for proteasome inhibition MG132 (Calbiochem). Control cells were treated with the same amount of solvent.

### Cloning of a plasmid to express HA-tagged G2E3

The G2E3 coding sequence was cloned into the vector pCGN-HA. The G2E3 coding sequence was amplified by PCR using pEGFP-C3-G2E3 [31] as a template. Primers were designed to include XbaI and KpnI restriction sites (5′-GCC GCC TCT AGA AAT GAA AGT AAA CCT GGT GAC-3′ and 5′-CGG CGG GGT ACC TTA ATG TCC AAT GTA ATG AG-3′), and these restriction enzymes were used to clone the PCR product into the vector. The correct sequence of pCGN-HA-G2E3 was verified by sequencing.

### High-content immunofluorescence microscopy

Cells were transfected and grown in 96-well imaging plates (Becton Dickison) and treated as indicated. After fixation with 4% paraformaldehyde in phosphate-buffered saline (PBS), cell permeabilization was performed using 0.5% Triton X-100 in PBS for 15 min. Cells were then incubated in blocking solution (0.1% Triton X-100 and 5% FCS in PBS) for 15 min. Immunofluorescence staining was performed using the following antibodies in blocking solution: mouse anti-phospho-H2AX (Ser319) antibody (Millipore) and Alexa Fluor-546 anti-mouse IgG (both Invitrogen/Life Technologies). Nuclei were stained with Hoechst 33342 (5 μg/ml; Invitrogen/Life Technologies).

Automated immunofluorescence microscopy was performed using the high-content imaging platform BD Pathway 855 System and the AttoVision image acquisition software (Becton Dickinson). All assays were performed in triplicates. For quantification of fluorescence intensity, single-cell-based image analysis was performed using the Hoechst signal to identify cell nuclei. For quantification, the average relative fluorescence intensity derived from γH2AX staining was determined per nucleus. The average fluorescence intensity per well was then calculated and background signals (determined by omitting the first antibody) were subtracted.

### High-content siRNA screening

Cells were reverse-transfected with a Silencer Select Ubiquitin Library (Ambion) containing siRNAs against 327 human ubiquitin ligases and 92 deubiquitinating enzymes with three different siRNAs per target gene on separate 96-well plates. The final siRNA concentration was 10 nM. Automated transfection of U2OS cells was performed using a Biomek 2000 laboratory automation workstation (Beckman Coulter). 32 h after transfection, cells were treated with 30 μM cisplatin for 16 h and fixed with 4% paraformaldehyde in PBS. Staining was done with Hoechst and the mouse anti-phospho-H2AX (Ser319) antibody (Millipore). High content automated microscopy and image analysis were performed as described above.

### Statistical analysis of the screen

Normalization of the screen data was performed using a robust z-score to correct for plate-to-plate variations [48]:

$$\text{z}=\frac{\text{sample value }-\text{ median of all samples}}{\text{median absolute deviation of all samples}}$$

*Sample value* refers to the average γH2AX fluorescence intensity per well. *Median of all samples* and *median absolute deviation of all samples* were calculated per plate. Using these values, the robust z-score was calculated for each well of the 96-well plate and thus for each siRNA. With the robust z-score, the effect of each siRNA on the accumulation of γH2AX was determined. After normalization of the data, a hit identification strategy was applied to take into account how many of the three different siRNAs per gene led to a significant decrease or increase of γH2AX fluorescence intensity. Hit identification was based on robust z-scores. Every siRNA with a robust z-score of ≥ 2 or ≤ −2 was considered as a hit. The sum of all three z-scores per gene was calculated and the genes were ranked according to this cumulative z-score. Candidates were identified if at least two siRNAs reached the threshold of ≥ 2 or ≤ −2.

### Statistical analysis

The number of independent experiments is stated with “n”. Error bars are depicted as standard deviation (SD) or standard error of the mean (SEM) as stated in the figure legend. An unpaired, two-tailed Student's t-test was used for the calculation of *p*-values. Asterisks are used to visualize *p*-values in the following way: ***p < 0.001, **p < 0.01, *p < 0.05, n.s. (not significant).

### Immunoblot analysis and antibodies

Proteins were separated by SDS-polyacrylamide gel electrophoresis and transferred to nitrocellulose membranes. For detection of proteins, membranes were incubated with antibodies diluted in 5% milk powder in Tris-buffered saline solution containing 0.1% Tween-20. Antibodies against phospho-groups were diluted in 5% BSA instead of milk powder. The following primary antibodies were used: goat anti-G2E3, mouse anti-hsc70, mouse anti-p53 (DO-1) (all Santa Cruz Biotechnology), rabbit anti-Caspase 3, rabbit anti-cleaved Caspase 3, mouse anti-Chk1, rabbit anti-PARP, rabbit anti-phospho-Chk1 (Ser317), rabbit anti-phospho-Chk2 (Thr68) (all Cell Signaling Technology), mouse anti-Chk2, mouse anti-Mdm2 (Ab-1), mouse anti-p21, mouse anti-PARP (all Calbiochem), mouse anti-actin (AC-15), rabbit anti-IgG (all Abcam), mouse anti-phospho-H2AX (Ser319) (Millipore), mouse anti-HA-tag (16B12, Covance), mouse anti-GFP (Clontech). Primary antibodies were detected by peroxidase-coupled secondary antibodies (Jackson ImmunoResearch Europe) using a Chemoluminescence Imaging System (Intas).

### Co-immunoprecipitation

For immunoprecipitation, six 15 cm cell culture dishes with adherent cells were used per sample. Cells were washed with phosphate-buffered saline (PBS) and harvested by scraping in lysis buffer (50 mM Tris-HCl, pH 7.5, 300 mM sodium chloride, 1% NP-40, 0.1% sodium deoxycholate, protease inhibitors). An equal amount of each protein lysate was incubated with antibodies overnight at 4°C (mouse anti-β-Galactosidase (Promega) and goat anti-G2E3 antibody (Santa Cruz)), followed by incubation with 25 μl protein G-sepharose (GE healthcare) for 1 h. As an additional control, antibodies were incubated with buffer only. The immune complexes were subjected to Western Blot analysis with goat anti-G2E3 antibody (Santa Cruz).

### Proliferation assay

For cell proliferation analysis, cells were reverse-transfected with siRNAs and grown in 12-well imaging plates (Corning). Cell proliferation was determined by measuring cell confluence daily for five days by bright-field microscopy with the *Celigo cell cytometer* (Cyntellect/Brooks Automation).

### Cell synchronization by double thymidine block

For synchronization of cell cultures, thymidine (Sigma-Aldrich) was added to the culture medium at a final concentration of 2 mM for 16 h. Thymidine was washed away by adding pre-warmed culture medium to the cells for 5 min five times. Subsequently, the cells were further incubated for 9 h. A second treatment with 2 mM thymidine for 16 h was performed. After washing away thymidine as before, the cells were released in fresh culture medium.

### Flow cytometry

For analysis by flow cytometry, cells were harvested, fixed in ethanol, and stained with propidium iodide (Sigma-Aldrich) at a final concentration of 30 μg/ml. Cell cycle profiles were obtained by quantifying the DNA content using the flow cytometer *Guava EasyCyte Plus system* and analyzed using *ModFit* software (Verity Software House).

### ssDNA assay

Detection of single-stranded DNA labeled with BrdU was done as described previously [28, 49]. Cells were reverse-transfected with siRNAs, incubated with 10 μM BrdU for 24 h and, under continued presence of BrdU, treated as indicated. Before fixation, the cells were preextracted at 4°C for 5 min with 0.5% Triton X-100, 20 mM Hepes, pH 7.5, 300 mM sucrose, 50 mM NaCl, and 3 mM MgCl_2_. Samples were stained by immunofluorescence as described earlier. BrdU was detected with mouse anti-BrdU antibody (RPN20AB; Amersham/GE Healthcare). The BrdU signal was quantified by high-content immunofluorescence microscopy as described above. Importantly, no DNA denaturing step (e.g. by HCl) was performed, leaving only pre-formed single-stranded DNA intermediates for detection.

### RNA Extraction and quantitative RT-PCR

For mRNA analysis, RNA was isolated from cells using TRIzol (Invitrogen/Life Technologies). The isolated RNA was reverse transcribed using 25U M-Mul V Reverse Transcriptase and random hexameric and oligo-dT primers. For analysis of cDNA samples, SYBR Green (Invitrogen/Life Technologies) was used for quantitative real-time PCR. The following primers were used: GAPDH, 5′-GAAGGTCGGAGTCAACGGATTTG-3′ and 5′-CAGAGATGATGACCCTTTTGGCTC-3′; G2E3, 5′-GGC GTG CCC CGA CGT ACA G-3′ and 5′-AGC AAG GTT CTG TGA GTC ACC AGG-3′; p21, 5′-TAG GCG GTT GAA TGA GAG G-3′ and 5′-AAG TGG GGA GGA GGA AGT AG-3′; p53, 5′-ATG GAG GAG CCG CAG TCA GAT C-3′ and 5′-GGG AGC AGC CTC TGG CAT TCT G-3′. Data were normalized to GAPDH. Relative gene expression was calculated by using the ΔΔCt method.

## SUPPLEMENTARY FIGURE AND TABLE





## References

[1] Jackson S, Bartek J (2009). The DNA-damage response in human biology and disease. Nature.

[2] Negrini S, Gorgoulis VG, Halazonetis TD (2010). Genomic instability—an evolving hallmark of cancer. Nat Rev Mol Cell Biol.

[3] Rabik CA, Dolan ME (2007). Molecular mechanisms of resistance and toxicity associated with platinating agents. Cancer Treat Rev.

[4] Wang D, Lippard S (2005). Cellular processing of platinum anticancer drugs. Nature reviews Drug discovery.

[5] Rezaee M, Sanche L, Hunting DJ (2013). Cisplatin enhances the formation of DNA single- and double-strand breaks by hydrated electrons and hydroxyl radicals. Radiation research.

[6] Mailand N, Bekker-Jensen S, Faustrup H, Melander F, Bartek J, Lukas C, Lukas J (2007). RNF8 ubiquitylates histones at DNA double-strand breaks and promotes assembly of repair proteins. Cell.

[7] Huen M, Grant R, Manke I, Minn K, Yu X, Yaffe M, Chen J (2007). RNF8 transduces the DNA-damage signal via histone ubiquitylation and checkpoint protein assembly. Cell.

[8] Doil C, Mailand N, Bekker-Jensen S, Menard P, Larsen D, Pepperkok R, Ellenberg J, Panier S, Durocher D, Bartek J, Lukas J, Lukas C (2009). RNF168 binds and amplifies ubiquitin conjugates on damaged chromosomes to allow accumulation of repair proteins. Cell.

[9] Huen M, Sy S, Chen J (2010). BRCA1 and its toolbox for the maintenance of genome integrity. Nature reviews Molecular cell biology.

[10] Higgins GS, Prevo R, Lee YF, Helleday T, Muschel RJ, Taylor S, Yoshimura M, Hickson ID, Bernhard EJ, McKenna WG (2010). A small interfering RNA screen of genes involved in DNA repair identifies tumor-specific radiosensitization by POLQ knockdown. Cancer Res.

[11] Cotta-Ramusino C, McDonald E, Hurov K, Sowa M, Harper J, Elledge S (2011). A DNA damage response screen identifies RHINO, a 9-1-1 and TopBP1 interacting protein required for ATR signaling. Science.

[12] Arora S, Bisanz KM, Peralta LA, Basu GD, Choudhary A, Tibes R, Azorsa DO (2010). RNAi screening of the kinome identifies modulators of cisplatin response in ovarian cancer cells. Gynecologic oncology.

[13] Paulsen R, Soni D, Wollman R, Hahn A, Yee M-C, Guan A, Hesley J, Miller S, Cromwell E, Solow-Cordero D, Meyer T, Cimprich K (2009). A genome-wide siRNA screen reveals diverse cellular processes and pathways that mediate genome stability. Molecular cell.

[14] Crawford D, Piwnica-Worms H (2001). The G(2) DNA damage checkpoint delays expression of genes encoding mitotic regulators. The Journal of biological chemistry.

[15] Beck H, Nahse V, Larsen MS, Groth P, Clancy T, Lees M, Jorgensen M, Helleday T, Syljuasen RG, Sorensen CS (2010). Regulators of cyclin-dependent kinases are crucial for maintaining genome integrity in S phase. J Cell Biol.

[16] Ritter J, Bielack SS (2010). Osteosarcoma. Annals of Oncology.

[17] Stad R, Ramos YF, Little N, Grivell S, Attema J, van Der Eb AJ, Jochemsen AG (2000). Hdmx stabilizes Mdm2 and p53. J Biol Chem.

[18] Li L, Deng B, Xing G, Teng Y, Tian C, Cheng X, Yin X, Yang J, Gao X, Zhu Y, Sun Q, Zhang L, Yang X, He F (2007). PACT is a negative regulator of p53 and essential for cell growth and embryonic development. Proc Natl Acad Sci U S A.

[19] Esser C, Scheffner M, Hohfeld J (2005). The chaperone-associated ubiquitin ligase CHIP is able to target p53 for proteasomal degradation. J Biol Chem.

[20] Waldman T, Kinzler KW, Vogelstein B (1995). p21 is necessary for the p53-mediated G1 arrest in human cancer cells. Cancer Res.

[21] Nijman SM, Huang TT, Dirac AM, Brummelkamp TR, Kerkhoven RM, D'Andrea AD, Bernards R (2005). The deubiquitinating enzyme USP1 regulates the Fanconi anemia pathway. Mol Cell.

[22] Huang TT, Nijman SM, Mirchandani KD, Galardy PJ, Cohn MA, Haas W, Gygi SP, Ploegh HL, Bernards R, D'Andrea AD (2006). Regulation of monoubiquitinated PCNA by DUB autocleavage. Nat Cell Biol.

[23] Butler LR, Densham RM, Jia J, Garvin AJ, Stone HR, Shah V, Weekes D, Festy F, Beesley J, Morris JR (2012). The proteasomal de-ubiquitinating enzyme POH1 promotes the double-strand DNA break response. Embo J.

[24] Brooks W, Helton E, Banerjee S, Venable M, Johnson L, Schoeb T, Kesterson R, Crawford D (2008). G2E3 is a dual function ubiquitin ligase required for early embryonic development. The Journal of biological chemistry.

[25] Cortez D, Guntuku S, Qin J, Elledge SJ (2001). ATR and ATRIP: partners in checkpoint signaling. Science.

[26] Liu Q, Guntuku S, Cui XS, Matsuoka S, Cortez D, Tamai K, Luo G, Carattini-Rivera S, DeMayo F, Bradley A, Donehower LA, Elledge SJ (2000). Chk1 is an essential kinase that is regulated by Atr and required for the G(2)/M DNA damage checkpoint. Genes Dev.

[27] Zhao H, Piwnica-Worms H (2001). ATR-mediated checkpoint pathways regulate phosphorylation and activation of human Chk1. Mol Cell Biol.

[28] Syljuasen RG, Sorensen CS, Hansen LT, Fugger K, Lundin C, Johansson F, Helleday T, Sehested M, Lukas J, Bartek J (2005). Inhibition of human Chk1 causes increased initiation of DNA replication, phosphorylation of ATR targets, and DNA breakage. Mol Cell Biol.

[29] Huarte M, Guttman M, Feldser D, Garber M, Koziol M, Kenzelmann-Broz D, Khalil A, Zuk O, Amit I, Rabani M, Attardi L, Regev A, Lander E, Jacks T, Rinn J (2010). A large intergenic noncoding RNA induced by p53 mediates global gene repression in the p53 response. Cell.

[30] Myers K, Gagou ME, Zuazua-Villar P, Rodriguez R, Meuth M (2009). ATR and Chk1 suppress a caspase-3-dependent apoptotic response following DNA replication stress. PLoS genetics.

[31] Brooks W, Banerjee S, Crawford D (2007). G2E3 is a nucleo-cytoplasmic shuttling protein with DNA damage responsive localization. Experimental cell research.

[32] Lavin M, Gueven N (2006). The complexity of p53 stabilization and activation. Cell death and differentiation.

[33] Horn HF, Vousden KH (2007). Coping with stress: multiple ways to activate p53. Oncogene.

[34] Meek DW, Anderson CW (2009). Posttranslational modification of p53: cooperative integrators of function. Cold Spring Harbor perspectives in biology.

[35] Momand J, Zambetti GP, Olson DC, George D, Levine AJ (1992). The mdm-2 oncogene product forms a complex with the p53 protein and inhibits p53-mediated transactivation. Cell.

[36] Kussie PH, Gorina S, Marechal V, Elenbaas B, Moreau J, Levine AJ, Pavletich NP (1996). Structure of the MDM2 oncoprotein bound to the p53 tumor suppressor transactivation domain. Science.

[37] Haupt Y, Maya R, Kazaz A, Oren M (1997). Mdm2 promotes the rapid degradation of p53. Nature.

[38] Kubbutat MH, Jones SN, Vousden KH (1997). Regulation of p53 stability by Mdm2. Nature.

[39] Thompson R, Eastman A (2013). The cancer therapeutic potential of Chk1 inhibitors: how mechanistic studies impact on clinical trial design. British journal of clinical pharmacology.

[40] Dent P, Tang Y, Yacoub A, Dai Y, Fisher PB, Grant S (2011). CHK1 inhibitors in combination chemotherapy: thinking beyond the cell cycle. Molecular interventions.

[41] Ma CX, Janetka JW, Piwnica-Worms H (2011). Death by releasing the breaks: CHK1 inhibitors as cancer therapeutics. Trends in molecular medicine.

[42] Zhang YW, Otterness DM, Chiang GG, Xie W, Liu YC, Mercurio F, Abraham RT (2005). Genotoxic stress targets human Chk1 for degradation by the ubiquitin-proteasome pathway. Mol Cell.

[43] Zhang YW, Brognard J, Coughlin C, You Z, Dolled-Filhart M, Aslanian A, Manning G, Abraham RT, Hunter T (2009). The F box protein Fbx6 regulates Chk1 stability and cellular sensitivity to replication stress. Mol Cell.

[44] Kilpinen S, Autio R, Ojala K, Iljin K, Bucher E, Sara H, Pisto T, Saarela M, Skotheim RI, Bjorkman M, Mpindi JP, Haapa-Paananen S, Vainio P, Edgren H, Wolf M, Astola J (2008). Systematic bioinformatic analysis of expression levels of 17,330 human genes across 9,783 samples from 175 types of healthy and pathological tissues. Genome biology.

[45] Hoeller D, Hecker C-M, Dikic I (2006). Ubiquitin and ubiquitin-like proteins in cancer pathogenesis. Nature reviews Cancer.

[46] Yang Y, Ludwig RL, Jensen JP, Pierre SA, Medaglia MV, Davydov IV, Safiran YJ, Oberoi P, Kenten JH, Phillips AC, Weissman AM, Vousden KH (2005). Small molecule inhibitors of HDM2 ubiquitin ligase activity stabilize and activate p53 in cells. Cancer cell.

[47] Jacobs C, Lyman G, Velez-Garcia E, Sridhar KS, Knight W, Hochster H, Goodnough LT, Mortimer JE, Einhorn LH, Schacter L (1992). A phase III randomized study comparing cisplatin and fluorouracil as single agents and in combination for advanced squamous cell carcinoma of the head and neck. Journal of clinical oncology : official journal of the American Society of Clinical Oncology.

[48] Birmingham A, Selfors L, Forster T, Wrobel D, Kennedy C, Shanks E, Santoyo-Lopez J, Dunican D, Long A, Kelleher D, Smith Q, Beijersbergen R, Ghazal P, Shamu C (2009). Statistical methods for analysis of high-throughput RNA interference screens. Nature methods.

[49] Kopper F, Bierwirth C, Schon M, Kunze M, Elvers I, Kranz D, Saini P, Menon MB, Walter D, Sorensen CS, Gaestel M, Helleday T, Schon MP, Dobbelstein M (2013). Damage-induced DNA replication stalling relies on MAPK-activated protein kinase 2 activity. Proc Natl Acad Sci U S A.

