# Supplementary material for: Screening analysis of ubiquitin ligases reveals G2E3 as a potential target for chemosensitizing cancer cells
Source: Oncotarget. 2014 Dec 15;6(2):617–32. doi: 10.18632/oncotarget.2710 (PMC4359243; doi:10.18632/oncotarget.2710)
Supplement: Supplementary file 1 [file oncotarget-06-617-s001.pdf]

SUPPLEMENTARY FIGURE AND TABLE

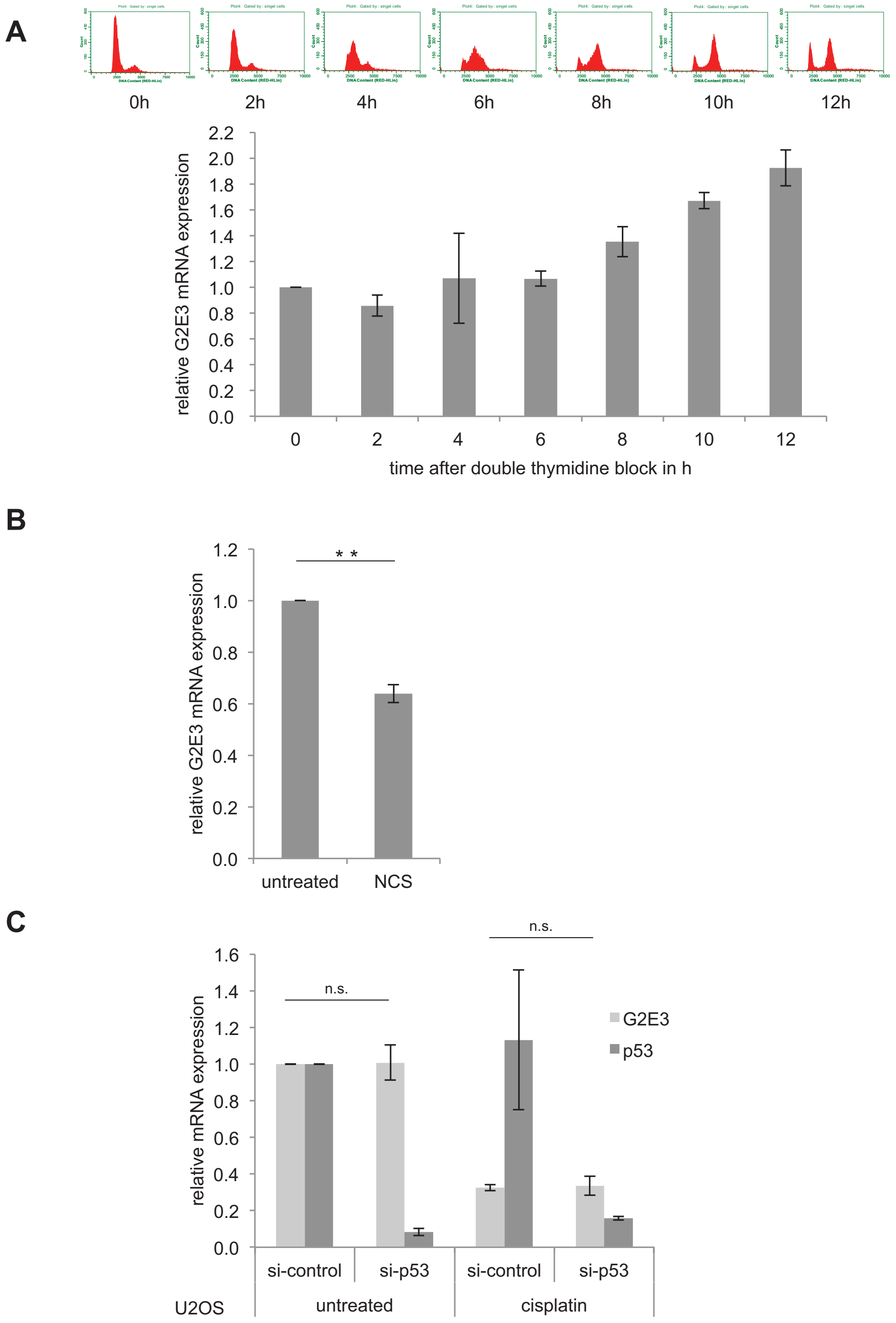

Supplementary Figure S7: Regulation of G2E3 mRNA levels. (A) G2E3 levels are at their maximum in the G2/M phase of the cell cycle. U2OS cells were arrested by a double thymidine block and then released. Cell cycle progression was monitored by flow cytometry of propidium iodide stained cells for 12 h after release from the block. The results in the upper panel are representative of three independent replicates. G2E3 mRNA levels were analyzed by quantitative RT-PCR at each time point. Results were normalized to the reference gene GAPDH. Data are represented as mean. Error bars represent the standard deviation (SD, $n = 3$). **(B) G2E3 mRNA levels are decreased after neocarzinostatin treatment.** U2OS cells were transfected with control-siRNA and either left untreated or treated with 150 ng/ml neocarzinostatin (NCS) for 2 h. Cells were harvested and G2E3 mRNA levels were analyzed by quantitative RT-PCR and normalized to the reference gene GAPDH. Data are represented as mean. Error bars represent the standard deviation (SD, $n = 3$). $^{**}p < 0.01$, (Student's *t*-test). **(C) Endogenous G2E3 mRNA levels are decreased after DNA damage, independent of p53.** U2OS cells were transfected with control- and p53-siRNA and treated with 30 µM cisplatin for 16 h. The cells were harvested and G2E3 and p53 mRNA levels were analyzed by quantitative RT-PCR as in B. Error bars represent the standard error of the mean (SEM, $n = 3$). n.s. = not significant (Student's *t*-test).

Supplementary Table S1: Results from the siRNA screen to identify modulators of the cellular response to cisplatin. U2OS cells were transfected with a library containing siRNAs against 327 human ubiquitin ligases and 92 deubiquitinating enzymes with three different siRNAs per target gene. The cells were treated with 30 µM cisplatin for 16 h, fixed and stained for γH2AX. Automated microscopy and image analysis was performed using the BD Pathway System. A robust z-score was assigned to each siRNA as a measure of H2AX phosphorylation. Genes are sorted by the average robust z-score of the three siRNAs. PC = positive control.
